# Idiopathic granulomatous mastitis: A case report and literature review

**DOI:** 10.1002/ccr3.7819

**Published:** 2023-08-25

**Authors:** Shiva Shabani, Bahman Sadeghi, Nader Zarinfar, Roham Sarmadian

**Affiliations:** ^1^ Clinical Research Development Unit of Ayatollah‐Khansari Hospital Arak University of Medical Sciences Arak Iran; ^2^ Department of Infectious Diseases, School of Medicine Arak University of Medical Sciences Arak Iran; ^3^ Department of Community Medicine, School of Medicine Arak University of Medical Sciences Arak Iran

**Keywords:** Brucella, idiopathic granulomatous mastitis

## Abstract

**Key Clinical Message:**

Idiopathic granulomatous mastitis (IGM) is a challenging chronic inflammatory disease in diagnosis with unknown etiology. Although the most appropriate treatment protocol has not yet been identified, prednisolone was used in our patient as an effective and practical choice in the treatment of IGM.

**Abstract:**

Idiopathic granulomatous mastitis (IGM) is a chronic inflammatory disease of the breast and mimics disorders such as breast cancer and breast abscess. Due to the uncommon of this disease, there is no definitive etiology, or treatment. A 38‐year‐old woman presented with a 3‐week history of painful right retro‐areolar mass. She had no history of breast trauma and a family history of breast cancer. She had a history of breastfeeding her second child for 12 months in the past year. Diagnostic tests and investigations led to the IGM diagnosis. Therefore, the patient was successfully treated with a course of corticosteroids, but after 2 months, during treatment, she developed Brucellosis. Despite the patient's Brucella infection and treatment with anti‐Brucella drugs, prednisolone as an anti‐inflammatory corticosteroid therapy was influential in the treatment of IGM.

## 1 INTRODUCTION

Idiopathic granulomatous mastitis (IGM) or granulomatous lobular mastitis is a rare chronic inflammatory disease of the breast in women.^1^ This disease commonly occurs shortly after a women's last pregnancy with a history of childbirth and breastfeeding that increases, especially in developing countries.^2^, ^3^ Despite the reports of an increase  in the prevalence of this disease in recent years, the cause of its etiopathogenesis remains little known and diversified.^3^ An autoimmune or hypersensitivity reaction is the most common hypothesis regarding the etiology of the disease. However, trauma to the epithelium of the mammary ducts and extravasation of milk or duct secretions to the connective tissue, hyperprolactinemia, oral contraceptives, or bacterial origin have been considered.^3^, ^4^

IGM usually presents with a unilateral or bilateral progressive painful breast lump. Patients with chronic IGM can develop fistulae, sterile abscesses, and nipple inversion.^5^ Bilateral IGMs have a higher relapse rate and more excellent resistance to medical therapies than unilateral IGMs.^6^ Histological evaluation applies to definite diagnosis while imaging methods differential diagnosis for breast cancer^3^ because abscesses can lead to being mistaken for breast cancer.^7^ Therefore, after causes must be considered, including breast cancer, autoimmune breast disease, and infection, the final diagnosis of IGM is often made.^4^, ^7^

**FIGURE 1:**
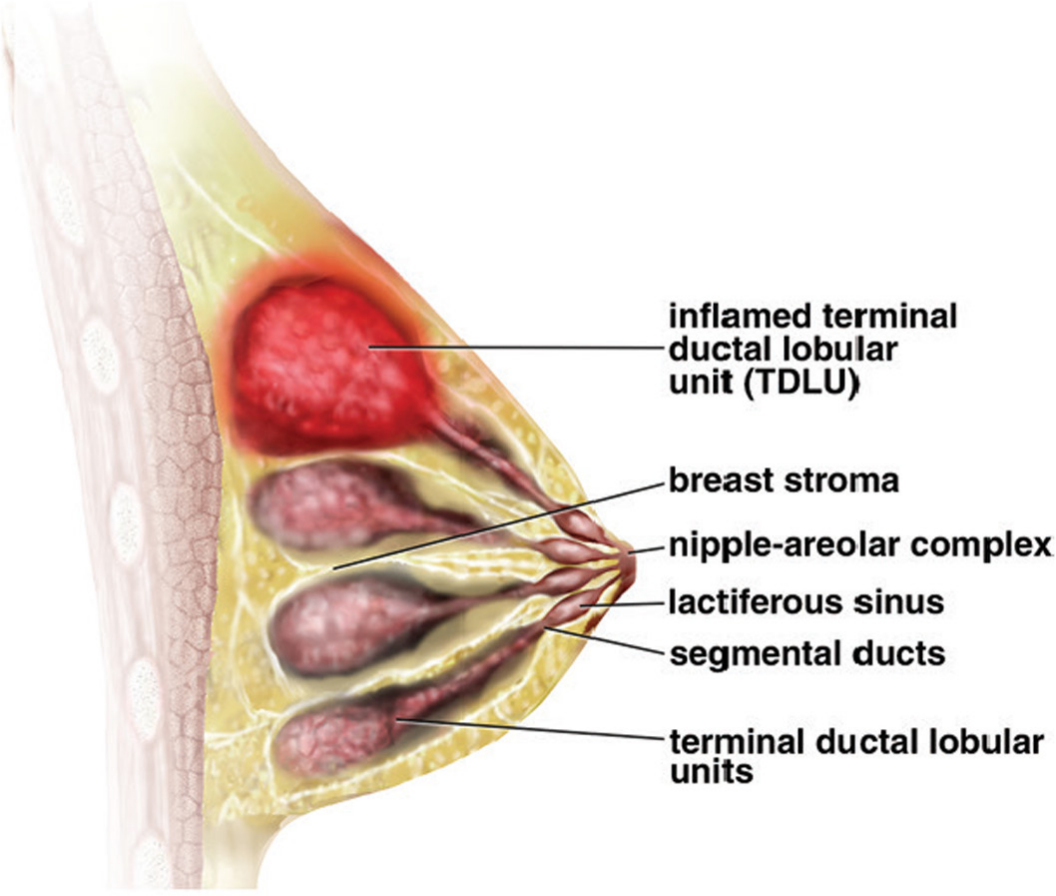
Illustration of typical clinical manifestations of IGM in the breast. A peripheral inflamed terminal ductal lobular unit with focal mass‐like properties is shown.^16^

Although the most appropriate treatment protocol has not yet been identified, some studies recommend surgical removal, while others suggest medical treatment such as antibiotics, corticosteroids, immunosuppressants, and anti‐inflammatory drugs.^3^ The results of our literature review  of IGM disease are summarized and exhibited in Table 1. Moreover, a graphic of the typical clinical manifestations of IGM in the breast is shown in Figure 1.

**TABLE 1:** Findings from the literature review for IGM

Age	Most women of childbearing age, several months to years after breastfeeding. Rare cases were reported in 11 and 80 years old.	5, 19
Mentioned etiopathogenesis	The etiopathogenesis is still unknown. Inflammation is a result of reaction to trauma, autoimmunity, and an infection such as *Corynebacterium* spp., and *Corynebacterium kroppenstedtii*. Metabolic or abnormal hormonal processes such as hyperprolactinemia. Lactation disorders	4, 8, 12, 17, 20, 21
Pathology	Noncaseating granulomas of a lobule‐centric pattern (multi‐nucleated giant cells, and epithelioid histiocytic located in the center of the lobules as well as neutrophils, lymphocytes, plasma cells, and a small number of eosinophils in the surrounding tissue) Lesions can be multifocal and form micro abscesses and vary in size.	4, 8, 12
Differential diagnosis	Idiopathic granulomatous lobular mastitis Periductal mastitis Fibrocystic changes, and Sclerosing lymphocytic lobulitis or diabetic mastopathy, Tuberculosis, fungal infections Malignancy	^10^
Imaging	Ultrasonographic findings: hypo‐echoic or heterogeneous mass were detected with or without tubular extensions. Magnetic resonance imaging (MRI) findings: focal or diffuse asymmetric signal intensity changes without significant mass effect were detected. On dynamic contrast‐enhanced MRI findings: IGM patients were detected with mass‐like or non‐mass‐like contrast enhancement, some of them with abscess positive. Mammographic presence of multiple contiguous iso‐dense masses, the reniform contour of axillary lymph nodes with the preserved fatty hilum. Contrast‐enhanced cone‐beam breast‐CT (CBBCT) findings: IGM mainly manifests is a non‐mass enhancement on CBBCT, with persistently enhancing or plateau TDC.	9, 22, 23, 24, 25
Treatment	Surgical Immunosuppressants, steroids, methotrexate, leflunomide, and antibiotics drugs Prolactin‐lowering medications	11, 12, 14, 15, 16, 20, 26, 27, 28

This study describes a patient with a breast lesion diagnosed as IGM, a rare chronic inflammatory disease, who 2 months after treatment with prednisolone presented symptoms of brucellosis.

## 2 CASE HISTORY/EXAMINATION

A 38‐year‐old woman was presented with a 3‐week history of a painful right retro‐areolar mass unresponsive to a course of antibiotics. She had no significant past medical history and had not used the oral contraceptive pill. The patient had no history of breast trauma and a history of breast cancer. Our case had two pregnancies in 14 and 2 last years. Also, she had a history of breastfed for her second child for 12 months in the previous year. Clinically, a unilateral firm right retro‐areolar breast mass was tender to palpation. The overlying skin was thickened and slightly warm. There was no associated nipple discharge or skin sinus.

## 3 DIFFERENTIAL DIAGNOSIS, INVESTIGATIONS, AND TREATMENT

**FIGURE 2:**
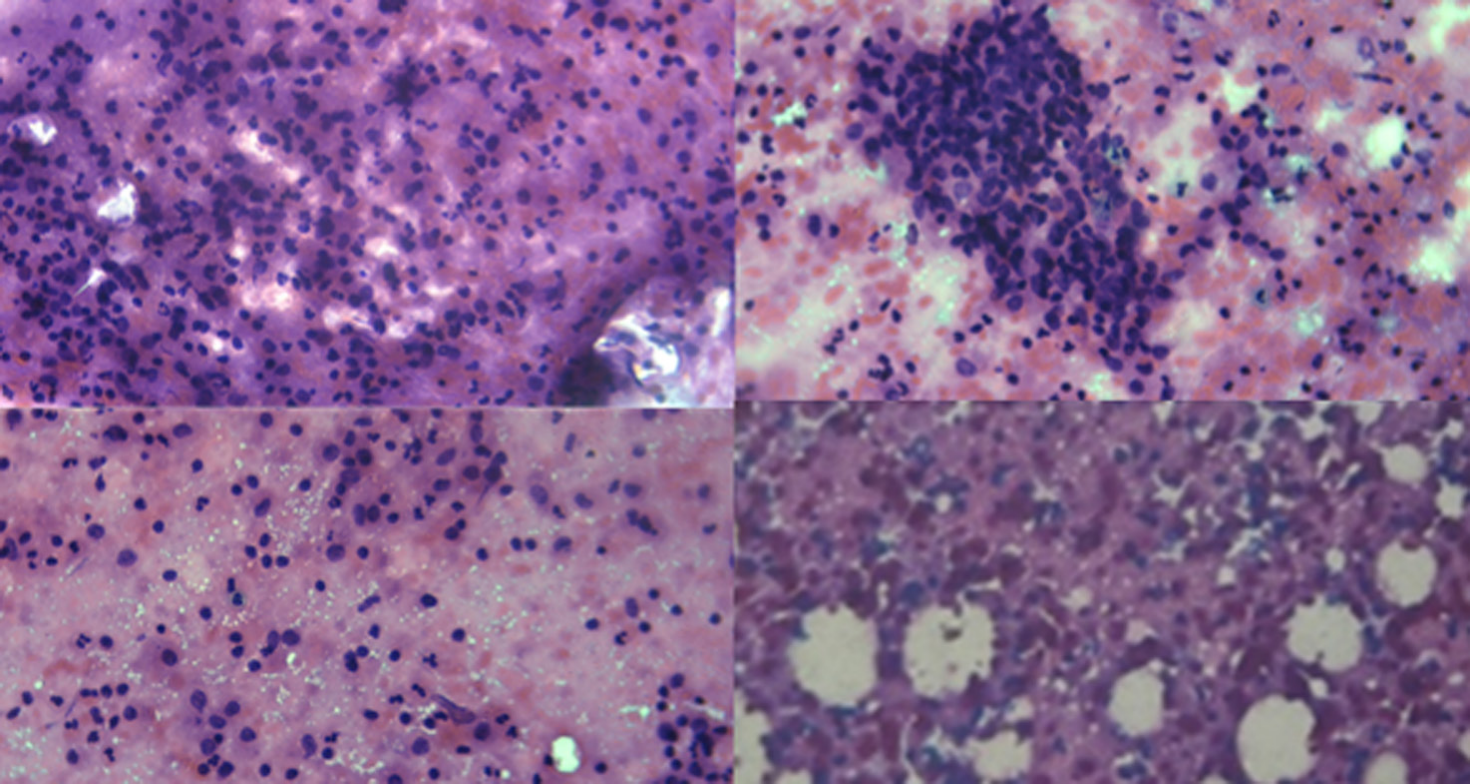
Pathological result for our patient with idiopathic granulomatous mastitis (IGM). Evaluating pathology indicated a number of ductal cells (1–2 hpf), red cells (15–20 hpf), frequent PMNs with macrophages, and epithelioid granuloma without the existence of malignant cells.

In continuation, our investigation by mammography for breast mass revealed bilateral moderately dense fibroglandular breast parenchyma in the right retro‐areolar region was a poorly defined area of increased density. Ultrasound revealed an irregularly outlined hypoechoic mass measuring 19 mm × 17 mm × 20 mm. Ultrasound‐guided core biopsy of the mass was performed. The biopsy demonstrated features of chronic granulomatous mastitis that was negative for malignant cells (Figure 2). Stains, in order to detect bacterial (Gram), fungal elements (PAS, Grocott's), and acid‐fast bacilli (Ziehl‐Neelsen), were negative. In continuation, negative results were obtained for the brucellosis serologic and autoimmune disease tests, including rheumatoid factor (RF): negative, anti‐cyclic citrullinated peptides (Anti‐CCP): 10 (0–30 U/mL), levels of angiotensin‐converting enzyme (ACE): 30 (<40 μg/L), anti‐double stranded DNA (anti‐dsDNA) test: 5 (<30.0 IU/mL), and erythrocyte sedimentation rate (ESR): 10 (0–15 mm/h).

The course of treatment, in this case, was started with prednisolone for 3.5 months. The dose of prednisolone in the first, second, and third months was 50, 25, and 25 mg per day, respectively. In the last 2 weeks, the prednisolone treatment was reduced and finally tapered to 12.5 and 5 mg. Although the patient was treated conservatively with steroids and showed good resolution of her symptoms within 1 month, after 2 months, the patient presented with symptoms of joint pain and headache. She was tested for Brucellosis and other tests because she lived in a Brucella endemic area and used local dairy products. The Brucella agglutination tests showed positive results with tetration Wright: 1/160, Coombs Wright: 1/160, and 2ME: 1/80. Therefore, she was treated with doxycycline and rifampin for 6 weeks, and prednisolone was continued as prescribed. The symptoms and signs of the recent illness (Brucellosis) improved.

## 4 OUTCOME AND FOLLOW‐UP

During the one‐year follow‐up, her symptoms and signs of IGM did not return.

## 5 DISCUSSION

IGM is a chronic benign breast disease observed in women of childbearing age, occurring within 5 years of the last delivery.^2^ Veyssiere et al. 1967 described IGM for the first time (Veyssiere 1967, as cited in Oze, 2022).^2^ The literature described that the prevalence of IGM is associated with race and region.^8^ Its etiopathogenesis remains unsolved, that diagnostic and therapeutic are challenging.

Mass lesions in IGM patients^9^ are of variable size, usually firm, tender, ill‐defined, and unilateral.^5^ In our patient, the lesion was retro‐areolar unilateral, ill‐defined, tender and warm in touch, and erythematous in appearance.

Often IGM is challenging to differentiate clinically and radiologically from infectious etiologies such as tuberculosis and fungal infections, and also from malignancy, thus posing a diagnostic dilemma.^10^ Our patient had received a course of antibiotic treatment due to a mistaken diagnosis of the infection. Also, she denied any previous disease and recent breast trauma. Finally, checking the breast for cancer and negative results for infectious tests led to accept the diagnosis of IGM.

There is no consensus about the optimal treatment for IGM.^11^ Several treatment modalities exist for patients with IGM that, to resolve its lesions completely, require more than one.^12^ A meta‐analysis illustrated that combining steroids and surgery in treating patients with IGM is better than only steroids. It even may lead to a lower rate of recurrence and side effects in these patients.^13^ In the last years, surgery has been avoided in most cases, introducing a more conservative medical approach.^14^ While some studies declare surgical in IGM patients with wide excision provides the best long‐term outcome.^15^, ^16^ In our case, the medical team did not recommend surgery, and corticosteroids were used to treat IGM. Unfortunately, after taking prednisolone for 2 months, she got Brucellosis. There is systemic immune dysregulation in patients with IGM, so alterations in T cells, NK, and NKT cells were reported.^17^ Moreover, prednisolone can weaken the immune system and make it easier to get infections. The literature demonstrates that corticosteroids increase the risk of severe conditions and some opportunistic infections.^18^

Collectively, IGM is a rare and challenging chronic inflammatory disease of the breast with unknown etiology and unusual manifestations. Although the most appropriate treatment protocol has not yet been identified, prednisolone was used in our patient as an effective and practical choice in the treatment of IGM. Although treatment with prednisolone was successful, infection with Brucella occurred during treatment in our patient.

## AUTHOR CONTRIBUTIONS

**Bahman Sadeghi:** Conceptualization; writing – original draft. **Shiva Shabani:** Project administration; writing – original draft. **Nader Zarinfar:** Investigation; writing – review and editing. **Roham Sarmadian:** Investigation; writing – review and editing.

## FUNDING INFORMATION

This research received no specific grant from any funding agency in the public, commercial, or not‐for‐profit sectors.

## CONFLICT OF INTEREST STATEMENT

All authors declare that they have no conflict of interest.

## ETHICS STATEMENT

The study protocol was approved by the Arak University of Medical Sciences Research Ethics Committee (IR.ARAKMU.REC.1401.227). All procedures performed in this study were in accordance with the 1964 Helsinki Declaration and its later amendments.

## CONSENT

Written informed consent was obtained from the patient to publish this report in accordance with the journal's patient consent policy.

## Data Availability

The data that support the findings of this study are available on request from the corresponding author.
